# Quality of life and patient satisfaction after implant‐based breast reconstruction with or without acellular dermal matrix: randomized clinical trial

**DOI:** 10.1002/bjs5.50324

**Published:** 2020-08-06

**Authors:** F. Lohmander, J. Lagergren, H. Johansson, P. G. Roy, J. Frisell, Y. Brandberg

**Affiliations:** ^1^ Department of Breast and Endocrine Surgery, Section of Breast Surgery, Karolinska University Hospital Stockholm Sweden; ^2^ Department of Surgery, Breast Centre, Capio St Görans Hospital Stockholm Sweden; ^3^ Department of Oncology–Pathology, Cancer Centre, Karolinska Institutet Stockholm Sweden; ^4^ Department of Molecular Medicine and Surgery Karolinska Institutet Stockholm Sweden; ^5^ Department of Breast Surgery Oxford University Hospitals NHS Foundation Trust Oxford UK

## Abstract

**Background:**

Acellular dermal matrix (ADM) in implant‐based breast reconstructions (IBBRs) aims to improve cosmetic outcomes. Six‐month data are presented from a randomized trial evaluating whether IBBR with ADM provides higher health‐related quality of life (HRQoL) and patient‐reported cosmetic outcomes compared with conventional IBBR without ADM.

**Methods:**

In this multicentre open‐label RCT, women with breast cancer planned for mastectomy with immediate IBBR in four centres in Sweden and one in the UK were allocated randomly (1 : 1) to IBBR with or without ADM. HRQoL, a secondary endpoint, was measured as patient‐reported outcome measures (PROMs) using three validated instruments (EORTC‐QLQC30, QLQ‐BR23, QLQ‐BRR26) at baseline and 6 months.

**Results:**

Between 24 April 2014 and 10 May 2017, 135 women were enrolled, of whom 64 with and 65 without ADM were included in the final analysis. At 6 months after surgery, patient‐reported HRQoL, measured with generic QLQ‐C30 or breast cancer‐specific QLQ‐BR23, was similar between the groups. For patient‐reported cosmetic outcomes, two subscale items, cosmetic outcome (8·66, 95 per cent c.i. 0·46 to 16·86; *P* = 0·041) and problems finding a well‐fitting bra (−13·21, −25·54 to −0·89; *P* = 0·038), yielded higher scores in favour of ADM, corresponding to a small to moderate clinical difference. None of the other 27 domains measured showed any significant differences between the groups.

**Conclusion:**

IBBR with ADM was not superior in terms of higher levels of HRQoL compared with IBBR without ADM. Although two subscale items of patient‐reported cosmetic outcomes favoured ADM, the majority of cosmetic items showed no significant difference between treatments at 6 months. Registration number: NCT02061527 ( www.clinicaltrials.gov).

## Graphical abstract

Implant‐based breast reconstruction (IBBR) with acellular dermal matrix (ADM) after mastectomy is common practice. A randomized trial was conducted to evaluate health‐related quality of life and patient‐reported cosmetic outcomes after ADM‐assisted IBBR in the setting of breast cancer treatment. Early results from this trial do not indicate a benefit for IBBR when supported with ADM.

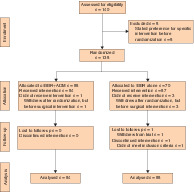

Acellular dermal matrix does not influence quality of life.

## Abstract (es)

**Antecedentes:**

Se ha propuesto la utilización de mallas dérmicas acelulares (*acellular dermal matrix*, ADM) en las reconstrucciones mamarias con prótesis (*implant’based breast reconstructions*, IBBR) como forma de mejorar los resultados estéticos. Se presentan los resultados a 6 meses de un ensayo aleatorizado, que evaluó si la IBBR con ADM proporcionaba mejor calidad de vida relacionada con la salud (*health’related quality of life*, HRQOL) y mejores resultados estéticos percibidos por la paciente en comparación con la IBBR convencional sin ADM.

**Resultados:**

Entre el 24 de abril de 2014 y el 10 de mayo de 2017, se consideraron 135 mujeres, de las que se incluyeron en el análisis final 64 con ADM y 65 sin ADM. A los 6 meses de la intervención, la HRQOL medida con los cuestionarios QLQ‐C30 (genérico) y QLQ‐BR23 (específico para el cáncer de mama) fue similar en los dos grupos. Con respecto a los resultados estéticos percibidos por la paciente, se obtuvieron mejores puntuaciones a favor de la ADM en dos sub‐escalas: “resultado estético” (8,66, i.c. del 95%, 0,46‐16,86, *P* = 0,041) y “problemas para encontrar un sujetador que se ajuste bien” −13,21 (i.c. del 95% −25, 54 a −0,89, *P* = 0,038), lo que representa una diferencia clínica pequeña‐moderada. No hubo diferencias significativas entre los dos grupos en ninguno de los otros 27 dominios medidos.

**Conclusión:**

No se pudo demostrar la superioridad de la IBBR con ADM mediante variables relacionadas con la calidad de vida. Aunque se obtuvieron mejores puntuaciones con la ADM en dos sub‐escalas de los PROMs, no hubo diferencias entre ambos tratamientos en la mayoría de las variables estéticas a los 6 meses.

## Introduction

Increased use of neoadjuvant chemotherapy, together with evolving oncoplastic techniques, has allowed for more breast‐conserving surgery^1^, but many women still undergo mastectomy^2^. Although reconstructions with autologous tissue have become more efficient, implant‐based breast reconstruction (IBBR) still remains the most frequently used method, particularly for reconstructions performed at the time of mastectomy^3^. As treatment for breast cancer commonly exposes women to distinct changes in their physical appearance, with radiotherapy and surgery significantly associated with poor body image, measuring surgical outcomes from the patients' perspective becomes a central component^4^. Furthermore, available data show that breast reconstruction after mastectomy can improve health‐related quality of life (HRQoL), and patient‐reported outcomes are also increasingly acknowledged by health providers as important tools in cancer treatment and care^4^, ^5^, ^6^, ^7^.

The introduction of acellular dermal matrix (ADM) in IBBRs aimed to create a more natural‐looking breast for women undergoing IBBR, as well as facilitating one‐stage breast reconstruction, and to improve HRQoL after mastectomy^8^, ^9^. The use of ADM and other types of mesh has in many institutions become common practice, with a range of different biological products and synthetic meshes now available. IBBR, supported with either a biological or synthetic mesh, is now the most widely used reconstruction method in the UK^10^. The initial responses to ADM in IBBR were overwhelmingly positive, with several studies reporting promising results with improved cosmesis, reduced need for tissue expanders, and less need for revisionary surgery for capsular contracture^11^. However, high‐quality evidence supporting these statements is still inconclusive, with data based mostly on small observational studies. Adding to this, data on potential benefits of HRQoL after ADM‐assisted IBBR are sparse^12^.

Several software tools are available on the market that attempt a more objective evaluation of cosmetic surgical outcomes, but there is no standardized objective tool for assessing cosmetic outcomes^13^. However, as body image and well‐being carry subjective experiences and perceptions, it becomes important that assessment of satisfaction after reconstructive surgery derives directly from the patient's own perspective, as patient‐perceived views do not necessarily align with physician‐reported scores^14^, ^15^.

Recently published data on harm have also raised concerns regarding the safety and effectiveness of IBBR supported with biological meshes^16^. A multicentre randomized trial in the Netherlands compared IBBR with ADM *versus* two‐stage IBBR without ADM. Outcomes from this trial revealed that single‐stage, direct‐to‐implant reconstructions augmented with ADM were associated with a significantly increased risk per breast of surgical complications and loss of implant, compared with two‐stage IBBR without ADM^16^. These results led to ethics committee suspension of further inclusion in the study. Furthermore, the suspension of ADM (Strattice™; Acelity, Branchburg, New Jersey, USA) by health authorities in France in 2015, because of high complication rates, raised further concerns regarding ADM and its potential harm. Data from the recently completed iBRA study^10^, a large prospective multicentre cohort study conducted in the UK, reporting on short‐term safety results after different types of breast reconstruction, did not reveal a higher incidence of reconstructive failure for mesh‐assisted IBBR.

The aim of this randomized trial was to evaluate a biological mesh (ADM) in the setting of breast cancer treatment in immediate IBBR, with the primary goal of comparing the number of reoperations between the study groups. Secondary outcomes were surgical complications (harm), quality of life and aesthetic considerations. In this trial, equal risk of implant loss was found between the groups at 6‐month follow‐up. However, IBBR with ADM was associated with more adverse outcomes requiring surgical interventions and reoperations. These early safety outcomes have been reported^17^. Here, HRQoL and patient‐reported satisfaction at 6 months after IBBR, a secondary endpoint in the trial, are described.

## Methods

Women with confirmed invasive or preinvasive breast cancer, planned for immediate IBBR with skin‐ or nipple‐sparing mastectomy, were eligible for inclusion. Exclusion criteria were: previous radiotherapy, neoadjuvant chemotherapy, smoking, BMI of 30 kg/m^2^ or above, predicted implant size smaller than 200 ml or greater than 600 ml, pregnant or lactating women, insulin‐dependent diabetes or any immunosuppressive disorder, allergy to porcine material or refusal to receive porcine material, or unable or unwilling to provide written informed consent. Written informed consent was obtained from all participants before any study‐related procedure was performed. The trial design has been described previously^17^.

The study protocol was approved by the Central Ethical Review Board in Stockholm (registration number 2012/1173‐31/1) and conducted according to the Declaration of Helsinki (Revised 2007). Separate ethical approval was obtained for the study centre in the UK (Integrated Research Application System project ID 150240).

### Randomization and masking

Potential participants were identified and recruited by local surgeons and enrolled from five different units in Sweden and the UK, and patients were assigned randomly (1 : 1) to the two types of breast reconstruction. A software module (Dynareg Systems; www.dynareg.se) was used to generate the randomization schedule. This process was also stratified between participating centres and in blocks of six, to ensure equal balance between treatment arms. Inclusion and exclusion criteria were verified automatically by the software. Randomization was performed by the research coordinator at each unit, after written informed consent had been obtained. Each participant was assigned a unique case number, recorded in a screening log kept locally. Physicians recruiting patients did not have access to the screening log. The study was open‐label, with both surgeons and patients being informed about the allocation result before the surgical procedure, but concealed to participants until they had completed two European Organization for Research and Treatment of Cancer (EORTC) questionnaires at baseline: the EORTC Quality of Life Questionnaire C30 (QLQ‐C30) and the EORTC QLQ Breast cancer module (QLQ‐BR23).

### Procedures

Participants were randomized to either immediate IBBR with ADM (Strattice™) and partial muscle coverage (ADM group) or immediate IBBR without ADM, using complete muscular coverage of the implant (control group). Allocation to treatment was done according to permuted block technique in units of six. Fixed‐volume implants or tissue expanders could be used in both groups, depending on tissue viability. After signing informed consent, participants were invited to complete the generic QLQ‐C30 and breast cancer‐specific QLQ‐BR23 questionnaires in the outpatient setting, or, if preferred, returning the questionnaires by prepaid post to the coordinating research nurse. The questionnaires were administered at baseline (before randomization) at the clinic. Follow‐up questionnaires were administered by post, including instructions for completion and a return envelope at three time points: 6, 12 and 24 months after reconstruction. A reminder was sent by the research nurse within 2–3 weeks.

All patients underwent skin‐ or nipple‐sparing mastectomy, and the reconstruction was performed by a breast or plastic surgeon, experienced with IBBR and familiar with the use of ADM. In the ADM group, the inferior insertion of the pectoralis major muscle was detached from the chest wall after mastectomy, and the ADM was sutured to its lower border and fixed along the inframammary fold, creating the implant pocket. The surgeon had the option of placing a definitive gel implant or using a tissue expander in both groups.

### Questionnaires

The EORTC QLQ‐C30 was developed to measure quality of life in patients with cancer in clinical trials^18^. It consists of 30 items comprising five functional scales: physical, emotional, social, role and cognitive functioning; and three symptom scales: fatigue, nausea and vomiting and pain. Six single items are also included: dyspnoea, insomnia, appetite loss, constipation, diarrhoea and financial difficulties. The final two items assess global health and overall quality of life. Most items are responded to on a 4‐point scale ranging from 1 (not at all) to 4 (very much). The two items assessing global health and overall quality of life are responded to in seven categories ranging from 1 (very poor) to 7 (excellent).

The EORTC QLQ‐BR23 comprises 23 questions, constituting five multi‐item scales assessing disease symptoms such as arm and breast symptoms, side‐effects of treatment (surgery, chemotherapy, radiotherapy and endocrine treatment), body image and sexual functioning^19^. In addition, sexual enjoyment, hair loss and future perspectives are measured by single items. The response format is the same as that for the core questionnaire.

The EORTC Breast Reconstruction Questionnaire (QLQ‐BRR26) was developed by the EORTC Quality of Life Group within the frame of EORTC HRQoL questionnaires, and assesses satisfaction with the results after breast reconstruction. It consists of 26 items, with scores ranging from 1 (not at all) to 4 (a lot), constituting seven scales: disease treatment/surgery related symptoms, problems finding a well‐fitting bra, sexuality, cosmetic outcome breast, cosmetic outcome donor site, satisfaction with reconstructed nipple, and problems with losing the nipple. The questionnaire was validated and tested for reliability in a set of women with breast cancer after breast reconstruction. The Swedish version was part of the development of this questionnaire^20^, ^21^.

### Statistical analysis

The sample size was calculated with respect to the primary trial endpoint, comparing the number of reoperations between the groups. A reoperation rate of 60 per cent in the control group and 30 per cent in the study group was estimated, observed over the course of 24 months from the primary procedure. In this paper, the aim was to evaluate HRQoL in ADM‐assisted IBBR, a secondary trial endpoint. No separate sample size calculation was performed for the secondary endpoints. Differences between treatment arms were estimated and tested using linear regression models, with subscale items as dependent variables and allocation groups as independent variables. Results from these models are presented as mean differences together with 95 per cent confidence intervals. Reported *P* values refer to Wald χ^2^ tests. Differences between the two treatment arms are presented as mean values, unadjusted at baseline measurement, and adjusted for baseline at the 6‐month measurement. The level of significance was set at 0·050.

For baseline characteristics, the *t* test was used for continuous variables and Fisher's exact test for categorical variables.

Results from the EORTC questionnaires were analysed according to the user instructions provided by the EORTC group. A standard EORTC scoring algorithm was used to transform scores linearly to ranges of 0–100. Clinically relevant differences were determined as follows: 5–9 as a small difference, 10–19 as moderate, and 20 or more as large^22^.

## Results

**Fig. 1:**
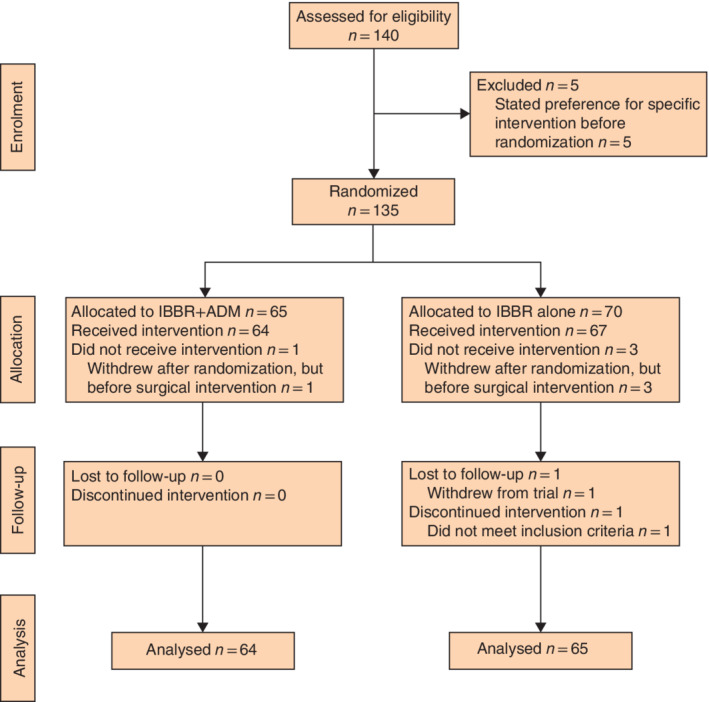
CONSORT diagram for the trial IBBR, implant‐based breast reconstruction; ADM, acellular dermal matrix.

Enrolment of participants took place between 24 April 2014 and 10 May 2017. A total of 135 women were randomized between the ADM group (65 patients) and the control group (70). Six patients were excluded from analysis after withdrawing from the study or not meeting the inclusion criteria, leaving 129 participants available for analysis, 64 in the ADM group and 65 in the control group (*Fig*. *1*).

**Fig. 2:**
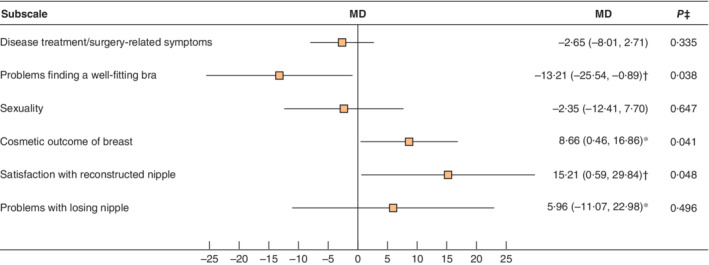
Forest plot illustrating mean differences in outcomes for the reconstruction‐specific EORTC‐BRR26 questionnaire at 6 months Mean differences (MDs) with crude estimates (no baseline) are shown with 95 per cent confidence intervals. *Small clinical difference; †moderate clinical difference^22^. ‡Wald test.

Baseline characteristics were similar in the two groups, with an overall mean(s.d.) age of 50·4(9·5) years and BMI of 23·4(2·7) kg/m^2^. Adjuvant radiotherapy was equally distributed between the groups, with 13 (20 per cent) in the ADM group and 19 (29 per cent) in the control group having started radiotherapy within the 6‐month follow‐up. Corresponding figures for number of patients who started chemotherapy within 6 months were 33 (52 per cent) and 30 (46 per cent) (*Table* *1*). At baseline, before randomization, there were no significant differences between the groups, measured with the generic QLQ‐C30 and breast cancer‐specific QLQ‐BR23 instruments (*Table* *2*). No statistically significant differences between randomization groups were found at the 6‐month follow‐up for these two instruments (*Table* *3*). For the breast reconstruction‐specific questionnaire (QLQ‐BRR26) at the 6‐month assessment, the subscale item cosmetic outcome breast yielded, on average, higher scores in the ADM group than in the control group (mean(s.d.) score 64(24) and 56(20) respectively; mean difference 8·66 (95 per cent c.i. 0·46 to 16·86, *P* = 0·041)) (*Table* *4* and *Fig*. *2*), corresponding to a small clinical difference. For the item Problems finding a well‐fitting bra, scores were higher in the ADM group compared with the control group, with a mean(s.d.) score of 27(33) and 40(33) respectively, with a mean difference of −13·21 (−25·54 to −0·89, *P* = 0·038) (*Table* *4* and *Fig*. *2*), a moderate clinical difference. The other domains in the QLQ‐BRR26 showed a tendency towards favouring ADM, but the differences were small and not significant.

**Table 1:** Baseline characteristics

	**Control (*n* = 65)**	**ADM (*n* = 64)**	***P*** **¶**
**Patient demographic data**			
Age (years)*	49·1(9·4)	51·8(9·5)	0·107#
BMI (kg/m^2^)*	23·0(2·7)	23·6(2·6)	0·201#
Invasive ductal cancer	28 (43)	32 (50)	0·482
Invasive lobular cancer	14 (22)	13 (20)	1·000
DCIS	23 (35)	17 (27)	0·342
Paget's disease of the breast	0 (0)	2 (3)	0·244
**Treatment‐related variables**			
Axillary surgery	63 (97)	64 (100)	0·496
Sentinel node only	57 (88)	52 (81)	0·341
Axillary lymph node clearance†	6 (9)	12 (19)	0·135
Nipple‐sparing mastectomy	32 (49)	26 (40)	0·378
Weight of mastectomy specimen (g)*	342·4(156·9)	358·4(161·5)	0·569#
Radiotherapy, initiated within follow‐up	19 (29)	13 (20)	0·309
Chemotherapy, initiated within follow‐up	30 (46)	33 (52)	0·599
**Allocation‐related variables** **‡**			
Direct to implant§	11 (17)	38 (59)	< 0·001
Implant volume (ml)*	255·9(46·9)	313·6(66·6)	< 0·001#
Expander volume (ml)*	383·6(83·2)	445·2(94·4)	< 0·001#
Intraoperative filling volume (ml)*	112·1(51·8)	149·8(64·3)	< 0·001#

Values in parentheses are percentages unless indicated otherwise;

* values are mean(s.d.). ADM, acellular dermal matrix; DCIS, ductal carcinoma *in situ*.

† With or without previous sentinel node.

‡ Variables dependent on allocation group.

§ Fixed‐volume implant at time of mastectomy.

¶ Fisher's exact test, except

# *t* test.

**Table 2:** Scores for EORTC QLQ‐C30 and QLQ‐BR23 questionnaires at baseline (before randomization)

	**Score**	
**Domain**	**Control (*n* = 65)**	**ADM (*n* = 64)**
**EORTC QLQ‐C30**	*n* = 61	*n* = 61
Functional scales		
Global health status	75(17)	72(21)
Physical functioning	98(6)	97(8)
Role functioning	90(19)	89(20)
Emotional functioning	68(23)	67(22)
Cognitive functioning	83(20)	86(16)
Social functioning	88(19)	90(18)
Symptom scales		
Fatigue	18(18)	15(15)
Nausea and vomiting	3(7)	1(6)
Pain	7(12)	7(13)
Dyspnoea	5(12)	9(18)
Insomnia	29(28)	33(29)
Appetite loss	10(18)	10(18)
Constipation	5(15)	4(11)
Diarrhoea	8(18)	3(12)
Financial difficulties	6(18)	6(19)
**EORTC QLQ‐BR23**	*n* = 59–61	*n* = 59–60
Functional scales		
Body image	85(20)	91(15)
Sexual functioning	32(25)	31(29)
Sexual enjoyment	72(24)	67(28)
Future perspective	42(33)	47(31)
Symptom scales		
Systemic therapy adverse effects	10(9)	9(8)
Breast symptoms	15(12)	14(17)
Arm symptoms	6(12)	4(10)
Hair loss	3(9)	3(11)

Values are mean(s.d.). ADM, acellular dermal matrix.

**Table 3:** Patient‐reported scores at 6‐month follow‐up for EORTC‐C30 and EORTC‐BR23 questionnaires

**Domain**	**Control (*n* = 65)** *	**ADM (*n* = 64)** *	**Mean difference** **†**	***P*** **§**
**EORTC QLQ‐C30**				
Functional scales				
Global health status	67 (8)	68 (10)	1·62 (−5·93, 9·16)	0·675
Physical functioning	88 (8)	90 (9)	2·89 (−1·74, 7·52)	0·224
Role functioning	74 (8)	76 (9)	3·71 (−6·32, 13·74)	0·470
Emotional functioning	74 (8)	79 (10)	4·99 (−3·09, 13·06)	0·229
Cognitive functioning	80 (8)	84 (10)	4·21 (−3·85, 12·27)	0·308
Social functioning	73 (8)	75 (10)	1·27 (−8·36, 10·90)	0·797
Symptom scales				
Fatigue	33 (8)	31 (9)	−2·24 (−11·34, 6·86)	0·631
Nausea and vomiting	4 (8)	5 (9)	0·42 (−3·08, 3·91)	0·816
Pain	21 (8)	16 (9)	−4·92 (−13·67, 3·84)	0·273
Dyspnoea	24 (8)	24 (9)	−1·23 (−12·06, 9·59)	0·824
Insomnia	33 (8)	32 (9)	−3·70 (−14·85, 7·46)	0·517
Appetite loss	7 (8)	7 (9)	−0·14 (−6·13, 5·84)	0·962
Constipation	18 (8)	9 (10)	−8·43 (−17·20, 0·35)‡	0·063
Diarrhoea	5 (8)	7 (10)	2·61 (−2·45, 7·68)	0·314
Financial difficulties	14 (8)	15 (10)	2·17 (−5·17, 9·51)	0·564
**EORTC QLQ‐BR23**				
Functional scales				
Body image	63 (8)	69 (10)	1·68 (−7·67, 11·02)	0·762
Sexual functioning	34 (11)	26 (14)	−5·05 (−13·29, 3·16)	0·231
Sexual enjoyment	65 (30)	67 (38)	3·64 (−7·74, 15·01)	0·533
Future perspective	47 (8)	53 (10)	0·67 (−10·80, 12·14)	0·909
Symptom scales				
Systemic therapy adverse effects	29 (8)	25 (10)	−3·76 (−11·01, 3·49)	0·312
Breast symptoms	26 (8)	17 (10)	−8·33 (−15·35, −1·31)	0·022
Arm symptoms	13 (8)	8 (10)	−2·62 (−8·25, 3·01)	0·364
Hair loss	25 (8)	22 (11)	−2·51 (−16·67, 11·66)	0·730

* Values are mean (range 0–100), with number of missing responses for each subscale in parenthesis;

† values in parentheses are 95 per cent confidence intervals.

‡ Small clinical difference^22^.

§ Wald test.

**Table 4:** Patient‐reported scores at 6 months for the breast reconstruction‐specific EORTC‐BRR26 questionnaire

**Domain**	**Control (*n* = 65)** *	**ADM (*n* = 64)** *	**Mean difference** **†**	***P*** **¶**
**EORTC QLQ‐BRR26**				
Disease treatment/surgery‐related symptoms	11 (8)	8 (9)	−2·65 (−8·01, 2·71)	0·335
Problems finding a well‐fitting bra	40 (9)	27 (9)	−13·21 (−25·54, −0·89)§	0·038
Sexuality	40 (8)	37 (10)	−2·35 (−12·41, 7·70)	0·647
Cosmetic outcome of breast	56 (8)	64 (9)	8·66 (0·46, 16·86)‡	0·041
Cosmetic outcome of donor site	n.o.	n.o.	n.o.	
Satisfaction with reconstructed nipple	50 (37)	65 (46)	15·21 (0·59, 29·84)§	0·048
Problems with losing nipple	35 (40)	41 (32)	5·96 (−11·07, 22·98)‡	0·496

* Values are mean (range 0–100), with number of missing responses for each subscale in parentheses;

† values in parentheses are 95 per cent confidence intervals. A higher score indicates higher satisfaction for Satisfaction with reconstructed nipple and Cosmetic outcome of breast; for all other domains a lower score indicates higher satisfaction.

‡ Small clinical difference;

§ moderate clinical difference^22^. ADM, acellular dermal matrix; n.o., no observations.

¶ Wald test.

## Discussion

Since ADM was introduced in IBBRs over a decade ago, discussion on the potential advantages has continued^16^, ^23^. With the goal of enhancing aesthetic outcomes after IBBR, and allowing for one‐stage breast reconstruction, HRQoL for women undergoing mastectomy would potentially improve^8^, ^24^, ^25^. However, few studies on ADM‐assisted IBBR have assessed patient‐reported outcomes and HRQoL, and data on cosmetic outcomes assessed from the patient's view are limited^26^. This RCT compared ADM‐assisted IBBR with conventional IBBR without ADM in the setting of breast cancer treatment. As a secondary endpoint, the study investigated whether ADM‐assisted IBBR provided higher HRQoL and patient satisfaction with the cosmetic outcome compared with conventional IBBR without ADM. At 6‐month follow‐up, overall HRQoL and patient‐perceived satisfaction with cosmetic outcomes, as measured with three different EORTC questionnaires, did not differ significantly between the two groups. Thus, no clear advantages could be confirmed with respect to HRQoL and self‐perceived cosmetic outcomes, when using ADM in IBBR.

The possible role of ADM in facilitating single‐stage reconstructions and improving cosmetic outcomes and hence quality of life is intuitively appealing. However, there remains a lack of prospective controlled data confirming earlier retrospective observational studies, and patient‐reported outcomes have not been assessed in most studies of ADM^26^. In a recently published paper from the Netherlands^27^, involving both therapeutic and risk‐reducing mastectomies, patient‐perceived satisfaction after ADM‐assisted IBBR was evaluated using Breast‐Q, a validated and condition‐specific tool for measuring outcomes after breast surgery. Findings from this RCT did not confirm a benefit for women who had IBBR augmented with ADM compared with the traditional two‐stage IBBR without ADM, with no statistically significant differences between the groups for HRQoL, sexual functioning and patient‐reported satisfaction with cosmetic results^27^. The conclusion was that further prospective studies were needed to evaluate the potential benefits of using ADM in IBBR compared with conventional IBBR. The trial from the Netherlands^27^ was designed exclusively to test the feasibility of performing single‐stage reconstructions, resulting in a high frequency of failure with loss of implant. Although this was adjusted for in a *post hoc* analysis, ADM still did not yield higher patient‐reported satisfaction, nor did the ADM group receive higher aesthetic scores assessed from photographs by a blinded panel of physicians^27^.

The Mastectomy Reconstruction Outcomes Consortium (MROC) Study^28^, a prospective observational study conducted at 11 sites in the USA and Canada, assessed long‐term outcomes between commonly used breast reconstruction techniques, evaluating complications and patient‐reported outcomes. These outcomes were measured using multiple instruments, including Breast‐Q and EORTC QLQ‐BR23. The results from this study^28^ did not show beneficial effects on cosmesis for IBBR augmented with ADM, and also reported higher rates of adverse events for ADM‐assisted IBBR.

In general, satisfaction with cosmesis can be influenced negatively by surgical complications. Although IBBR with ADM was not related to a higher incidence of reconstructive failure within the follow‐up time of 6 months, the overall number of surgical complications requiring reoperation was higher in the ADM group than in controls^17^. Whether this imbalance had an impact on HRQoL outcomes in the present study has not been explored.

In two prospective observational single‐centre studies^12^, ^29^, HRQoL was assessed after ADM‐assisted IBBR, also using Breast‐Q. In contrast to the present study, both of these studies reported favourable scores for ADM, although neither used comparison groups for reference.

Equally, as with the RCT from the Netherlands^27^, the present study did not confirm any significant improvement for the ADM group with regard to patient‐reported satisfaction of cosmetic outcomes.

In this study, scores related to the subscales Cosmetic outcome and Problems finding a well‐fitting bra in the QLQ‐BRR26 module showed, both statistically and clinically, differences in favour of the ADM group. However, these differences were small and applied to only two of 29 analysed domains. This interpretation could also have been confounded by the higher proportion of women reconstructed with ADM having a one‐stage, direct‐to‐implant procedure, compared with the control group, where a higher proportion of reconstructions were performed using an expander–implant. Perhaps more importantly, within the 6‐month follow‐up, none of the participants had an implant exchange^17^. Scores for the reconstruction‐specific module BRR26 were comparable to results reported by Bai and colleagues^30^, who also used EORTC‐BRR26 to evaluate patient‐perceived aesthetics. This prospective follow‐up study^30^ assessed long‐term psychosocial outcomes for women with an increased hereditary risk for breast cancer having risk‐reducing mastectomy and IBBR without ADM.

The present study has several limitations. First, adjuvant radiotherapy is a known risk factor for complicating the postoperative course^31^. As the authors elected to present data with a follow‐up of 6 months, the impact that further revisional surgery after radiotherapy, as well as chemotherapy, might have had on patient satisfaction with cosmetic outcomes and HRQoL could have been underestimated. However, as an equal number of patients had initiated radiotherapy within the follow‐up time, the factor of adjuvant therapy was not taken into consideration in this study. Second, of the 29 subscale items tested, significant group differences in favour of ADM‐assisted IBBR were noted for only two subscales. Although both of these domains specifically measure satisfaction with cosmetic outcomes, the differences were small to moderate, with broad confidence intervals. In addition, the pragmatic design of the trial, allowing for expander implants to be placed in both groups, makes it difficult to assess early results before any implant exchanges have occurred. Although permanent expanders (Becker 35™; Mentor, Santa Barbara, California, USA) were frequently applied in this study, many patients at the authors' institution elect to have these exchanged for fixed‐volume implants as a second‐stage procedure, probably because revisional surgery frequently follows adjuvant radiotherapy. Added to this, single‐stage reconstructions with fixed‐volume implants could also be interpreted as a more ‘final’ procedure by the patient, resulting in a bias towards ADM‐assisted IBBR when assessing HRQoL. Third, the reconstruction‐specific tool EORTC QLQ‐BRR26 used in this study was tested for reliability in 2017. However, there is to date only one published paper^30^ evaluating results after breast reconstructions using this survey. This makes it difficult to put the present results into a broader context. Equally, use of the Breast‐Q questionnaire in the Dutch RCT^27^ prevents direct comparison of data between these two randomized trials.

Since the present trial was initiated, the practice of IBBR has evolved, with the introduction of prepectoral techniques with or without a biological or synthetic mesh^32^. The surgical technique used in the present control group, of fully covering the implant with muscle, has in some institutions now become an almost outdated type of reconstructive method. However, with the prepectoral implant placement being repopularized today, it is also worth mentioning that the previous subcutaneous implant placement used in the 1980s, with the subsequent shift to complete muscle coverage in the 1990s, was driven by high complication and capsular contracture rates, but also led to an inferior aesthetic outcome^33^.

This appears to be the first randomized trial to assess HRQoL and patient‐reported satisfaction prospectively after ADM‐assisted IBBR in the setting of breast cancer treatment, without incorporating risk‐reducing mastectomy. Early results indicate that ADM was not associated with a higher HRQoL. For patient‐reported satisfaction with cosmesis, a possible advantage was noted for ADM, but considering the short follow‐up time and modest differences for only two subscale items, this should be interpreted cautiously.

## References

[1] Regionala Cancer Centrum . *National Quality Register for Breast Cancer* https://www.cancercentrum.se/samverkan/cancerdiagnoser/brost/kvalitetsregister/ [accessed 1 February 2020].

[2] Kummerow KL , Du L , Penson DF , Shyr Y , Hooks MA . Nationwide trends in mastectomy for early‐stage breast cancer. JAMA Surg 2015; 150: 9–16. 25408966 10.1001/jamasurg.2014.2895

[3] American Society of Plastic Surgeons . 2017 *Cosmetic Plastic Surgery Statistics* . https://www.plasticsurgery.org/documents/News/Statistics/2017/plastic‐surgery‐statistics‐report‐2017.pdf [accessed 1 July 2020].

[4] Falk Dahl CA , Reinertsen KV , Nesvold I‐L , Fosså SD , Dahl AA . A study of body image in long‐term breast cancer survivors. Cancer 2010; 116: 3549–3557. 20564138 10.1002/cncr.25251

[5] Porter ME . What is value in health care? N Engl J Med 2010; 363: 2477–2481. 21142528 10.1056/NEJMp1011024

[6] Chao L‐F , Patel KM , Chen SC , Lam HB , Lin CY , Liu HE *et al* Monitoring patient‐centered outcomes through the progression of breast reconstruction: a multicentered prospective longitudinal evaluation. Breast Cancer Res Treat 2014; 146: 299–308. 24951266 10.1007/s10549-014-3022-7

[7] Black N. Patient reported outcome measures could help transform healthcare. BMJ 2013; 346: f167. 23358487 10.1136/bmj.f167

[8] Salzberg CA . Nonexpansive immediate breast reconstruction using human acellular tissue matrix graft (AlloDerm). Ann Plast Surg 2006; 57: 1–5. 16799299 10.1097/01.sap.0000214873.13102.9f

[9] Salzberg CA , Ashikari AY , Berry C , Hunsicker LM . Acellular dermal matrix‐assisted direct‐to‐implant breast reconstruction and capsular contracture: a 13‐year experience. Plast Reconstr Surg 2016; 138: 329–337. 27064232 10.1097/PRS.0000000000002331

[10] Potter S , Conroy EJ , Cutress RI , Williamson PR , Whisker L , Thrush S *et al*; iBRA Steering Group; Breast Reconstruction Research Collaborative. Short‐term safety outcomes of mastectomy and immediate implant‐based breast reconstruction with and without mesh (iBRA): a multicentre, prospective cohort study. Lancet Oncol 2019; 20: 254–266. 30639093 10.1016/S1470-2045(18)30781-2 PMC6358590

[11] Cassileth L , Kohanzadeh S , Amersi F . One‐stage immediate breast reconstruction with implants: a new option for immediate reconstruction. Ann Plast Surg 2012; 69: 134–138. 21734545 10.1097/SAP.0b013e3182250c60

[12] Headon H , Kasem A , Manson A , Choy C , Carmichael AR , Mokbel K . Clinical outcome and patient satisfaction with the use of bovine‐derived acellular dermal matrix (SurgiMend™) in implant based immediate reconstruction following skin sparing mastectomy: a prospective observational study in a single centre. Surg Oncol 2016; 25: 104–110. 27312036 10.1016/j.suronc.2016.03.004

[13] Keshtgar MR , Williams NR , Bulsara M , Saunders C , Flyger H , Cardoso JS *et al* Objective assessment of cosmetic outcome after targeted intraoperative radiotherapy in breast cancer: results from a randomised controlled trial. Breast Cancer Res Treat 2013; 140: 519–525. 23877341 10.1007/s10549-013-2641-8

[14] Potter S , Harcourt D , Cawthorn S , Warr R , Mills N , Havercroft D *et al* Assessment of cosmesis after breast reconstruction surgery: a systematic review. Ann Surg Oncol 2011; 18: 813–823. 20972633 10.1245/s10434-010-1368-6

[15] Gill TM , Feinstein AR . A critical appraisal of the quality of quality‐of‐life measurements. JAMA 1994; 272: 619–626. 7726894

[16] Dikmans RE , Negenborn VL , Bouman MB , Winters HA , Twisk JW , Ruhé PQ *et al* Two‐stage implant‐based breast reconstruction compared with immediate one‐stage implant‐based breast reconstruction augmented with an acellular dermal matrix: an open‐label, phase 4, multicentre, randomised, controlled trial. Lancet Oncol 2017; 18: 251–258. 28012977 10.1016/S1470-2045(16)30668-4

[17] Lohmander F , Lagergren J , Roy PG , Johansson H , Brandberg Y , Eriksen C *et al* Implant based breast reconstruction with acellular dermal matrix: safety data from an open‐label, multicenter, randomized, controlled trial in the setting of breast cancer treatment. Ann Surg 2019; 269: 836–841. 30308615 10.1097/SLA.0000000000003054

[18] Aaronson NK , Ahmedzai S , Bergman B , Bullinger M , Cull A , Duez NJ *et al* The European Organization for Research and Treatment of Cancer QLQ‐C30: a quality‐of‐life instrument for use in international clinical trials in oncology. J Natl Cancer Inst 1993; 85: 365–376. 8433390 10.1093/jnci/85.5.365

[19] Sprangers MA , Groenvold M , Arraras JI , Franklin J , te Velde A , Muller M *et al* The European Organization for Research and Treatment of Cancer breast cancer‐specific quality‐of‐life questionnaire module: first results from a three‐country field study. J Clin Oncol 1996; 14: 2756–2768. 8874337 10.1200/JCO.1996.14.10.2756

[20] Thomson HJ , Winters ZE , Brandberg Y , Didier F , Blazeby JM , Mills J . The early development phases of a European Organisation for Research and Treatment of Cancer (EORTC) module to assess patient reported outcomes (PROs) in women undergoing breast reconstruction. Eur J Cancer 2013; 49: 1018–1026. 23063353 10.1016/j.ejca.2012.09.021

[21] Winters ZE , Balta V , Thomson HJ , Brandberg Y , Oberguggenberger A , Sinove Y *et al* Phase III development of the European Organization for Research and Treatment of Cancer Quality of Life Questionnaire module for women undergoing breast reconstruction. Br J Surg 2014; 101: 371–382. 24474151 10.1002/bjs.9397

[22] Osoba D , Rodrigues G , Myles J , Zee B , Pater J . Interpreting the significance of changes in health‐related quality‐of‐life scores. J Clin Oncol 1998; 16: 139–144. 9440735 10.1200/JCO.1998.16.1.139

[23] Potter S , Browning D , Savović J , Holcombe C , Blazeby JM . Systematic review and critical appraisal of the impact of acellular dermal matrix use on the outcomes of implant‐based breast reconstruction. Br J Surg 2015; 102: 1010–1025. 26109277 10.1002/bjs.9804

[24] Breuing KH , Warren SM . Immediate bilateral breast reconstruction with implants and inferolateral AlloDerm slings. Ann Plast Surg 2005; 55: 232–239. 16106158 10.1097/01.sap.0000168527.52472.3c

[25] Macadam SA , Lennox PA . Acellular dermal matrices: use in reconstructive and aesthetic breast surgery. Can J Plast Surg 2012; 20: 75–89. 23730154 10.1177/229255031202000201 PMC3383551

[26] Negenborn VL , Dikmans RE , Bouman MB , Wilschut JA , Mullender MG , Salzberg CA . Patient‐reported outcomes after ADM‐assisted implant‐based breast reconstruction: a cross‐sectional study. Plast Reconstr Surg Glob Open 2018; 6: 1654. 10.1097/GOX.0000000000001654 PMC5865927 29616167

[27] Negenborn VL , Young‐Afat DA , Dikmans RE , Smit JM , Winters HA , Griot JP *et al* Quality of life and patient satisfaction after one‐stage implant‐based breast reconstruction with an acellular dermal matrix *versus* two‐stage breast reconstruction (BRIOS): primary outcome of a randomised, controlled trial. Lancet Oncol 2018; 19: 1205–1214. 30104147 10.1016/S1470-2045(18)30378-4

[28] Sorkin M , Qi J , Kim HM , Hamill JB , Kozlow JH , Pusic AL *et al* Acellular dermal matrix in immediate expander/implant breast reconstruction: a multicenter assessment of risks and benefits. Plast Reconstr Surg 2017; 140: 1091–1100. 28806288 10.1097/PRS.0000000000003842 PMC5705287

[29] El Hage Chehade H , Headon H , Wazir U , Carmaichael AR , Choy C , Kasem A *et al* Nipple‐sparing mastectomy using a hemi‐periareolar incision with or without minimal medial–lateral extensions; clinical outcome and patient satisfaction: a single centre prospective observational study. Am J Surg 2017; 213: 1116–1124. 27523925 10.1016/j.amjsurg.2016.04.016

[30] Bai L , Arver B , Johansson H , Sandelin K , Wickman M , Brandberg Y . Body image problems in women with and without breast cancer 6–20 years after bilateral risk‐reducing surgery – a prospective follow‐up study. Breast 2019; 44: 120–127. 30743225 10.1016/j.breast.2019.01.013

[31] Lin AM , Christensen JM , Liao EC , Cetrulo CL Jr , Smith BL , Austen WG Jr *et al* Postmastectomy radiation therapy on permanent implants or tissue expanders: which is better? Ann Surg 2019; doi: 10.1097/SLA.0000000000003670 [Online ahead of print]. 31714307

[32] Chatterjee A , Nahabedian MY , Gabriel A , Macarios D , Parekh M , Wang F *et al* Early assessment of post‐surgical outcomes with pre‐pectoral breast reconstruction: a literature review and meta‐analysis. J Surg Oncol 2019; 117: 1119–1130. 10.1002/jso.24938 29346711

[33] Slade CL . Subcutaneous mastectomy: acute complications and long‐term follow‐up. Plast Reconstr Surg 1984; 73: 89–90. 6691079

